# Severe complications after mesh migration following abdominal hernial repair: report of two cases and review of literature

**DOI:** 10.3205/iprs000135

**Published:** 2019-05-17

**Authors:** Giulia Manzini, Doris Henne-Bruns, Michael Kremer

**Affiliations:** 1 Department of General and Visceral Surgery, University Hospital of Ulm, Germany; 2 Department of General and Visceral Surgery, Kantonsspital Aarau, Switzerland

**Keywords:** ventral hernia, incisional hernia, mesh migration, mesh erosion

## Abstract

**Background:** Migration of mesh after ventral and incisional hernia repair is a rare but well described complication. The aim of our work is to present two cases of mesh migration after incisional hernia repair and to review the current literature.

**Methods:** We describe the two cases of mesh migration that occurred at our department. Additionally, we performed a systematic literature search.

**Results:** In both cases we observed a mesh migration with formation of an entero-cutaneous fistula that required surgical therapy. In the literature search we found a total of 16 publications dealing with mesh migration after incisional (n=14) and ventral hernia (n=2) repair in adult patients (15 case reports and one retrospective study). In 9 out of 15 patients (54%) who presented with mesh migration or erosion, a polypropylene mesh was responsible for this complication.

**Conclusions:** Mesh migration after abdominal hernia repair is rare, the only available retrospective study reports a rate of 2.7%. The ability of polypropylene mesh to migrate into hollow viscera is well known and confirmed both by our data and the results of the literature review. As the incidence of mesh erosion/migration is significantly lower than the recurrence rate after hernia repair without mesh, up to now, no better alternative exists for the treatment of abdominal wall hernia than mesh augmentation.

## Zusammenfassung

**Hintergrund:** Netzwanderung nach chirurgischer Versorgung von ventralen- oder Narbenhernien ist eine seltene, aber gut beschriebene Komplikation. Ziel der hier vorliegenden Arbeit ist die Präsentation von zwei Fällen und eine Übersicht der aktuellen Literatur.

**Methoden:** Zunächst beschreiben wir zwei Fälle von Netzwanderung in unserer Abteilung. Zusätzlich führten wir eine systematische Literaturrecherche durch.

**Ergebnisse:** In beiden Fällen von Netzwanderung ging die Komplikation mit Ausbildung einer entero-kutanen Fistel einher, so dass eine erneute chirurgische Therapie notwendig wurde. Im Rahmen der Literaturrecherche fanden wir insgesamt 16 Publikationen über Netzwanderung nach Narbenhernien- (n=14) und ventraler Hernienversorgung (n=2) in Erwachsenen (15 Einzelfallberichte und eine retrospektive Studie). Bei 9 von 15 Patienten (54%) mit Netzwanderung oder Erosion war ein Polypropylene-Netz für die Ausbildung der Komplikation verantwortlich.

**Schlussfolgerung:** Netzwanderung nach abdomineller Hernienversorgung ist selten, die einzig vorliegende retrospektive Studie beschreibt eine Rate von 2.7%. Die Möglichkeit der Wanderung von Polypropylen-Netzen in Hohlorgane ist bekannt und sowohl durch unsere Fallberichte als auch die Literaturrecherche bestätigt. Da die Inzidenz von Netzwanderung und Erosion signifikant niedriger als das Auftreten von Rezidiven nach Hernienverschluss ohne Netz ist, existieren bisher keine besseren Alternativen für die Behandlung von Bauchdeckenhernien als die Netzverstärkung.

## Introduction

The use of meshes for the repair of ventral and incisional abdominal hernia is a common practice in order to provide tension-free repair of the fascial defect [1] with a low rate of recurrence. However, mesh repair increases the risk of infection [2] and can result in mesh erosion, fistula formation, [3] and rarely, mesh migration [4]. Migration of mesh after any hernia repair can be subdivided into primarily mechanical reasons or secondarily due to erosion of surrounding tissue [5]. Primary mechanical migrations are displacements of the mesh by either inadequate fixation or probably external displacing forces. Secondary migrations are slow and gradual movements of the mesh through trans-anatomical planes. They have been observed after foreign body reaction-induced erosion and are increasingly being recognised in the literature [5]. Mesh migrations seem to be independent to a great extent from the nature of mesh (bio) material and from the type of fixation of the mesh, if fixed at all. Thus, the mesh initially may get displaced but later erode into adjacent tissue [5]. Clinical presentation is therefore varying depending on the site of the migration [5] and comprise the occurrence of an enteric fistula [6], [7], [8], [9], the erosion of small bowel [10] or colon with chronic abdominal pain [11], [12] or obstruction symptoms [13]. The aim of our work is to report about two patients who presented with entero-cutaneous fistula caused by mesh migration after incisional hernia repair and to present an overview of the existing literature.

## Case descriptions

### Case 1 

A 68-year-old female presented in 3/2018 with a high output small bowel fistula after several laparotomies and incisional hernia repair. 

In 11/2008, an en bloc resection of ovaries, adnexa and uterus combined with lymphadenectomy and anterior rectal resection with protective ileostomy was performed because of a stage four cervix carcinoma. The operation was followed by chemotherapy and abdominal radiation. The gastrointestinal continuity was reconstruction 4/2009. 

In 9/2016 the patient presented with an acute abdomen due to a spontaneous ileum perforation. The emergency operation revealed massive adhesions and a perforation of the small bowel in the lower abdomen, necessitating a small bowel resection. The postoperative course was eventful leading to an open abdomen, repeated abdominal lavage therapy and an ileostomy. 10/2016 the abdomen could be closed by inlay mesh implantation (Symbotex composite mesh 25x20 cm). The high output ileostomy required parenteral feeding via a venous catheter. 

In 9/2017, the patient was readmitted for reconstruction of the small bowel continuity after continuous weight loss and signs of malnutrition. An end-to-end ileo-ileostomy was performed after extended adhesiolysis. However, the patient again developed a leakage at the anastomotic side and required several operative interventions. She was discharged with a high output small bowel fistula and a short bowel syndrome in 12/2017 under complete parenteral nutrition.

**Figure 1:**
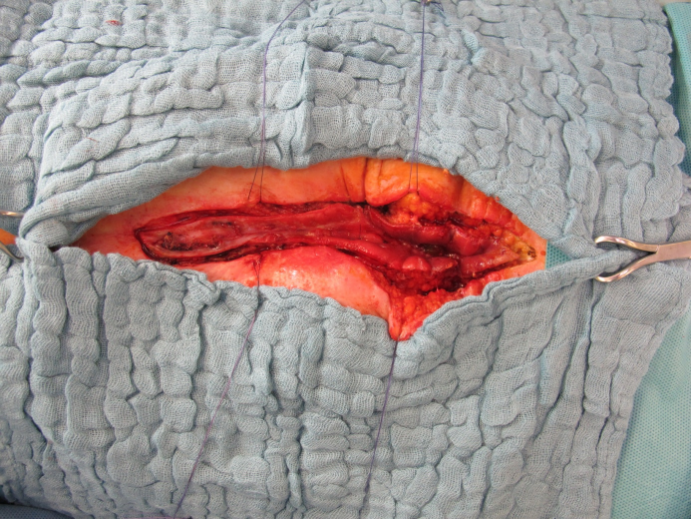
First case. Intraoperative situs of the incised skin with the high output enterocutanous fistula

**Figure 2:**
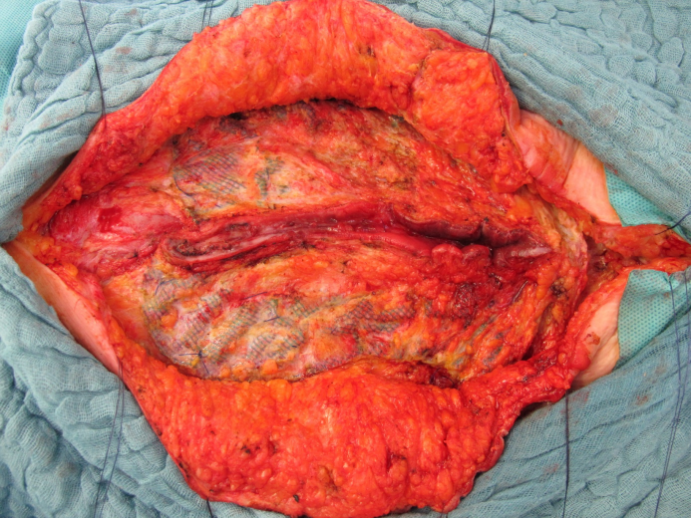
Implanted mesh with central enterocutanous fistula

**Figure 3:**
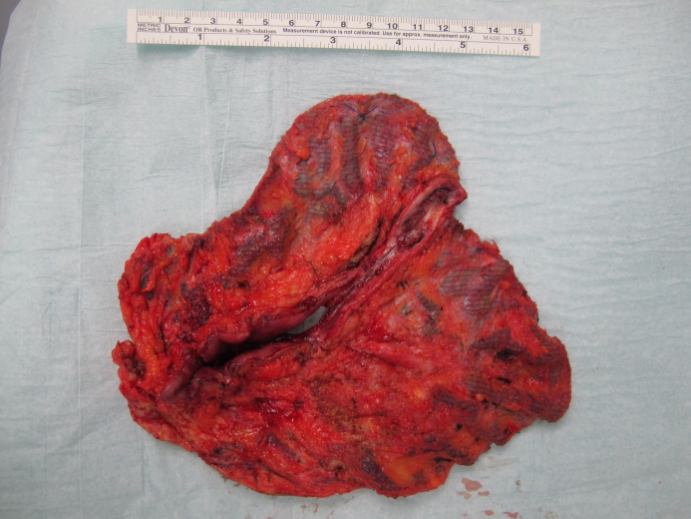
Figure 3 : En-bloc exzised mesh with the enterocutanous fistula centrally

In 3/2018 the patient developed a second small bowel fistula and signs of infection at the midline incision and the abdominal wall. Parts of the mesh became visible. After antibiotic pretreatment, re-laparotomy with extirpation of the mesh, adhesiolysis, spare resection of the small bowel including the fistulas, ileo-ileostomy and reconstruction of the abdominal wall was performed in 4/18 (Figure 1 (Fig. 1), Figure 2 (Fig. 2), Figure 3 (Fig. 3)) . From the intraoperative findings it was clear that the mesh eroded the small bowel and caused the fistulas.

**Figure 4:**
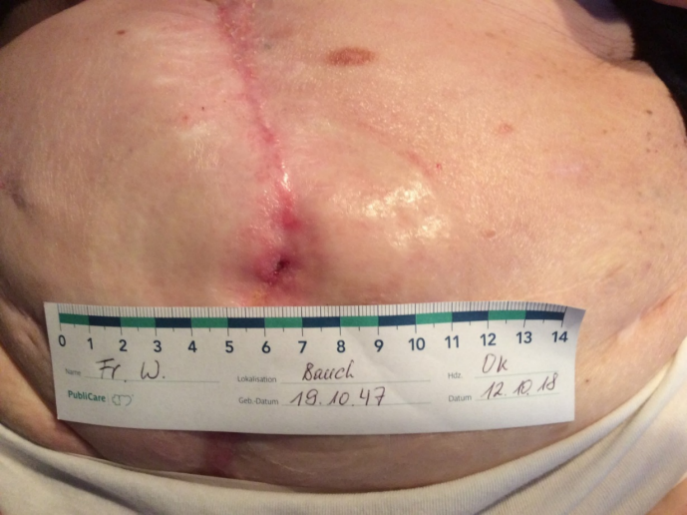
Stable low output entero-cutaneous fistula 24 months after resection of the migrated mesh. No evidence of hernia-recurrence (with written consent of the patient)

The postoperative course was again eventful and resulted in fistula formation, but fortunately only a small low-output fistula developed. After 3 months of intensive wound-therapy, the patient was able to handle the low-output fistula like a small stoma and presented in 10/2018 with complete oral intake and a secretion of less than 5cc per day (Figure 4 (Fig. 4)). 

### Case 2 

**Figure 5:**
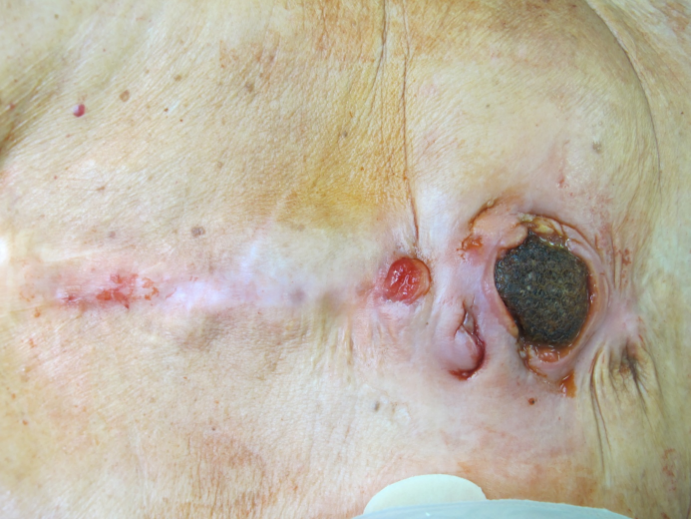
Second case. Enterocutanous fistula with migrated mesh

A 90-year-old male patient presented in 5/2018 with a high output small bowel fistula secreting through a 4x4 cm perforation of the skin and through the underlying mesh (Figure 5 (Fig. 5)). 

The patient had a history of transverse colon resection in 12/2008 (colon carcinoma T3, N0, MO), insufficiency of the anastomosis with repeated operative interventions and creation of a terminal ileostomy. In 7/2009 a hernia of the abdominal wall was repaired using a Proceed mesh. 10/2010 he underwent resection of liver metastasis (segment 2 and 3). The patient showed several comorbidities like hypertension, Parkinson’s disease and nephropathy that required hemodialysis.

**Figure 6:**
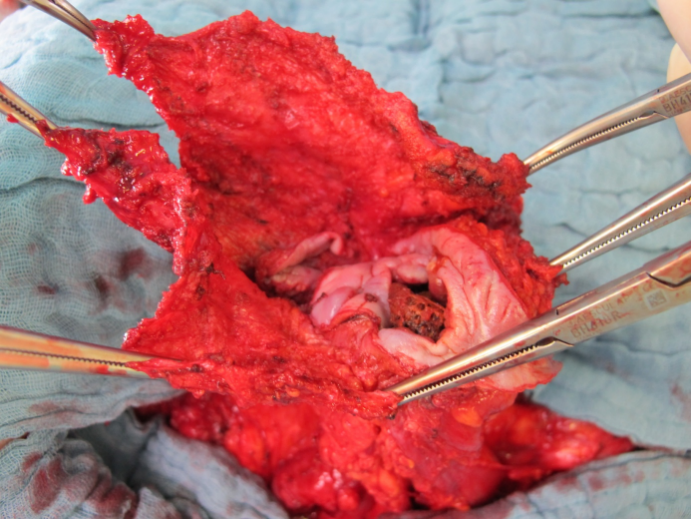
Intraoperative picture of excision of migrated mesh with fistula

**Figure 7:**
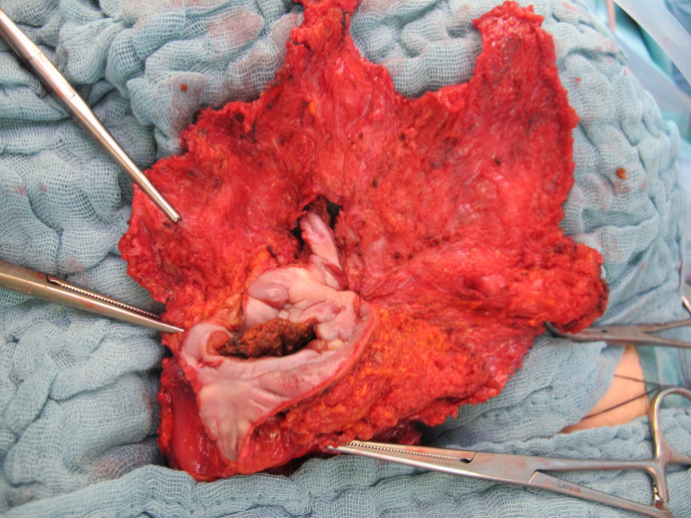
Intraoperative picture of excision of migrated mesh with fistula

**Figure 8:**
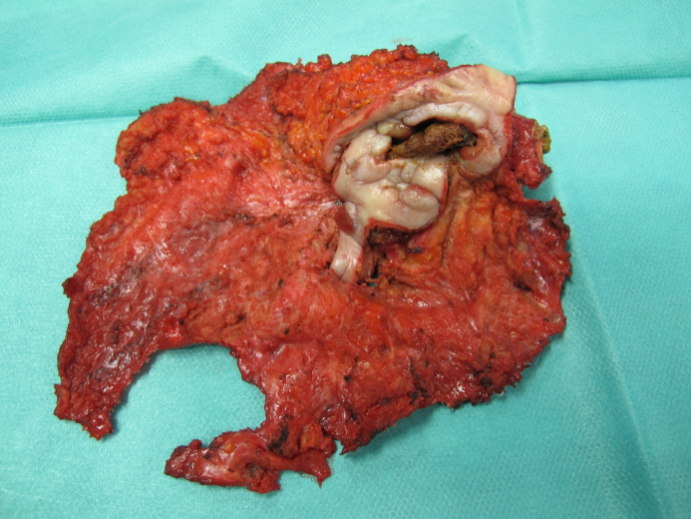
Excised mesh with the enterocutanous fistula

An exploratory laparotomy with removal of the mesh and en bloc resection of two segments of the small bowel was performed (Figure 6 (Fig. 6), Figure 7 (Fig. 7), Figure 8 (Fig. 8)). The closure of the abdominal wall without alloplastic material could only be achieved by doubling of the dermis after removal of the epithelium. He experienced a complicated clinical course with several re-explorations because of insufficiency of the small bowel anastomoses. Finally, again, an enterocutaneous fistula developed. The general condition of the patient decreased continuously and the patient died one month after the operation.

## Review of the literature

We performed a systematic literature search with the key words “mesh migration after hernia repair” and “enteric fistula after hernia repair” and “bowel perforation after mesh migration” on October 7th 2018. We screened the database Medline, Cochrane and Pub Med. Inclusion criteria were each type of publication in English describing the migration of mesh after ventral or incisional hernia repair. All publications dealing with inguinal hernia repair were excluded. We identified a total of 21 abstracts [1], [6], [7], [8], [9], [10], [11], [12], [13], [14], [15], [16], [17], [18], [19], [20], [21], [22], [23], [24], [25] which could be assessed for further analysis, 5 of them were available only in abstract form and were therefore not considered [21], [22], [23], [24], [25]. A total of 16 publications were included, one retrospective study [10] and 15 case reports [1], [6], [7], [8], [9], [11], [12], [13], [14], [15], [16], [17], [18], [19], [20]. One case report included a literature review [17]. Table 1 (Tab. 1) shows the 16 included publications.

## Discussion

Mesh migration after hernia repair is a rare complication. Tollens et al. [26] performed a retrospective analysis of the Ventralex hernia patch used for incisional and ventral hernia repair in 176 patients aiming to evaluate complications. No migrations and/or erosions were observed in the cohort after a mean follow-up of 49 months (range 13–70 months). Recurrence of herniation was observed in 12 patients (8.9%) [26]. In the retrospective study of Ratajczak et al. [10] a total of 77 patients underwent abdominal hernia surgery with mesh implantation. Migration was observed in two patients (2.6%), both after implantation of a polypropylene mesh. 

Meshes evoke similar inflammatory reaction simultaneously in both gastro-intestinal and genito-urinary tracts close to hernia repairs [27], possibly resulting in isolated enteric fistulas or entero-vesical fistulas. According to Yolen [28], intra-abdominal foreign bodies like meshes transmigrate into the bowel by initiating an inflammatory reaction. The foreign body is then encapsulated by omentum, and along with the inflammatory reaction may create an opening into a hollow organ assisted by the peristaltic movement of the bowel [28]. 

Investigations have shown that in incisional hernia repair direct exposure of the intestines to absorbable and non-absorbable biomaterials could result in their adhesion to the bowel [29]. Similarly, in inguinal hernia repairs, cases of small bowel obstruction secondary to mesh plug migration [30] and intra-peritoneal mesh migration [31] have been reported. 

The method of fixation may affect migration rates by altering the tensile strenght and degree of movement of the mesh. The size, shape and positioning of the mesh may also be an important factor, as well as the nature of the biomaterial, as it affects the extent and degree of interaction with the surrounding tissue [12]. In particular, the nature of the mesh material may induce erosion. Polypropylene meshes offer long-term stability, but can induce acute inflammation with infiltration by granulocytes and macrophages. Polyglactin mesh causes less inflammation than other meshes. Composite meshes, which are made of multifilament polypropylene and polyglactin, are manufactured with different materials on each surface, strategically positioning the different surfaces to selectively impede or promote tissue ingrowth. The more inert mesh material is intended to prevent adhesions with the underlying viscera, and multiple studies have demonstrated its effectiveness [16]. 

Our patients had an abdominal wall reconstruction with either a composite or proceed mesh.

The removal of the mesh and closure of the high output fistulas was in both cases without any alternatives. Patient 1 suffered from malnutrition under complete parenteral nutrition and experienced several septic episodes with removal and reimplantion of the venous catheter system. Furthermore, she developed a phlegmone of the abdominal wall close to the perforation sites.

Patient 2 developed an enterocutaneous fistula close to the ileostomy. The high output fistula caused problems regarding the oral intake of his medication. Furthermore, a save cover of this second stoma was impossible, leading to a constant exposure of the skin to small bowel secretions.

The ability of polypropylene mesh to migrate into hollow viscera is well known and confirmed both by the results of the literature review of Picchio et al. [18] and by our work. In 9 out of 15 patients (54%) who showed a mesh migration or erosion, a polypropylene mesh was responsible for this complication. 5 patients had a composite mesh and one patient a polyester mesh. For the 2 remaining patients the mesh material was not reported. The question if biodegradable/complete dissolving meshes are better remains open. 

The risk of migration is much higher if the mesh is placed intraperitoneally in direct contact to the viscera. The adjunct of barriers, such as expanded polytetrafluoroethylene (ePTFE), does not avoid visceral adhesions and consequent possible erosion [32]. 

Our review of literatur revealed that meshes causing erosions were placed either intraperitoneally (n=4), as an inlay (n=1), in onlay (n=1) and underlay (n=1) position. In one case, the mesh was placed supraperitoneally, not described in any more detail. In 8 cases, the position could not be determined exactly by the description. 

Beside the position of mesh, probably more factors which increase the risk of wound infection (comorbidities like smoking or diabetes, chronic inflammatory disease) can increase the risk of mesh erosion/migration because of the inflammatory response. 

## Conclusion

In conclusion, although mesh migration presents a potentially life threatening long term complication, we have to reflect that the incidence of mesh erosion/migration is significantly lower than the recurrence rate after hernia repair without mesh. Actually no better alternative exists for the treatment of incisional and ventral hernia than mesh augmentation.

## Notes

### Competing interests

The authors declare that they have no competing interests.

## Figures and Tables

**Table 1:**
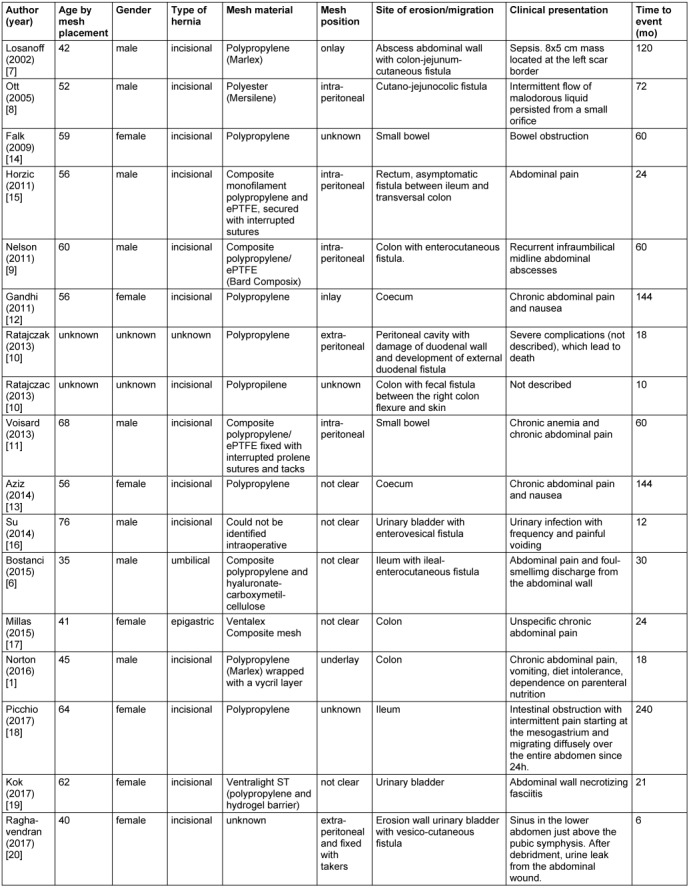
Literature on mesh migration/erosion after ventral (n=2) and incisional hernia repair (n=15)
